# Successful Treatment of Cisplatin Overdose with Plasma Exchange

**DOI:** 10.1155/2010/802312

**Published:** 2010-03-11

**Authors:** Yasuhiro Yamada, Yoshiaki Ikuta, Kisato Nosaka, Nobutomo Miyanari, Naoko Hayashi, Hiroaki Mitsuya, Hideo Baba

**Affiliations:** ^1^Department of Hematology & Infectious Diseases, Kumamoto University School of Medicine, Kumamoto 860-8556, Japan; ^2^Department of Gastroenterological Surgery, Kumamoto University School of Medicine, Kumamoto 860-8556, Japan

## Abstract

Accidental cisplatin overdose has been occurring with an increasing frequency due to expanding usage of the agent. However, the optimal strategy to treat such patients remains to be established. Here, we report a case of large cisplatin overdose, successfully managed by plasma exchange, intravenous hydration, granulocyte colony-stimulating factor (G-CSF) administration, and other supportive care. A 67-year-old man with esophageal carcinoma received a large cisplatin overdose of 240 mg/m^2^, when he received adjuvant therapy following subtotal esophagectomy. On day 4, he experienced frank cisplatin toxicities and emergency plasma exchange was initiated. With 7 cycles of plasma exchange, the cisplatin concentration decreased from 2,350 to 110 ng/mL. Severe bone marrow suppression with high fever ensued on day 10, which was successfully treated with G-CSF and antibiotics. Despite moderate hearing sense reduction, he recovered without significant complications. Immediate plasma exchange with hydration and other care was efficacious in quickly lowering cisplatin concentrations.

## 1. Introduction

Cis-diamminedichloroplatinum (II) (cisplatin) represents one of the most widely used and effective antineoplastic agents. The heavy metal platinum causes interstrand cross-linking of DNA, thereby preventing tumor cell proliferation [1]. Preclinical data suggest that cisplatin has a steep dose-response relationship for ovarian cancer and other tumors [2]. However, despite vigorous intravenous hydration and mannitol treatment, acute nephrotoxicity and chronic renal damage often occur after administration of therapeutic doses of cisplatin, 100 to 120 mg/m^2^ per one cycle of chemotherapy [3]. In particular, higher doses of cisplatin due to accidental overdose have been reported to cause nephrotoxicity, neurotoxicity, ototoxicity, gastrointestinal disturbances, and severe myelosuppression [4]. Although there are reports describing that patients receiving massive cisplatin overdose were successfully rescued [4–8], the optimal strategy to treat overdosed patients remains to be established.

Here, we report a 67-year-old man who suffered an accidental cisplatin overdose of 240 mg/m^2^. Although the patient was left with moderately reduced sense of hearing, he ultimately recovered without significant complications with plasma exchange combined with intravenous hydration, G-CSF administration, and other supportive care.

## 2. Case Report

**Figure 1:**
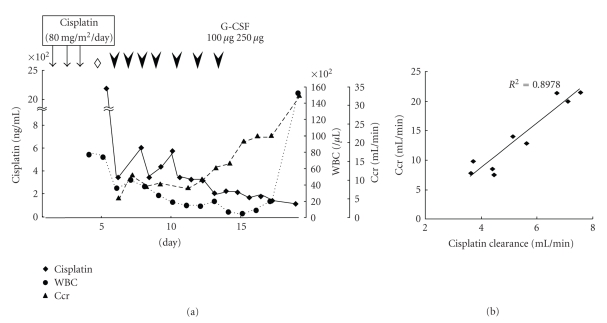
*Plasma cisplatin concentrations, leukocyte counts, Ccr values and platinum clearance values. *(a) An open diamond and arrow heads denote for dialysis and plasma exchange, respectively. (b) Note that cisplatin clearance approximately correlated with Ccr. WBC: white blood cell, G-CSF: granurocyte-colony-stimulating factor, Ccr: creatinine clearance.

A 67-year-old man was diagnosed with stage II esophageal carcinoma (T1N2M0). Endoscopic examination showed a white plaque lesion spreading from 35 to 37 cm from incisors after spraying of Lugol's iodine solution. No spread beyond the adventitia was apparent with both computed tomography (CT) and positron emission tomography examinations. However, metastatic lymph node involvements in regions I and III were noted. Histopathology revealed well-differentiated squamous cell carcinoma. He underwent subtotal esophagectomy and was diagnosed to be at postoperative stage IIIa (pT3N3M0). He subsequently received postoperative adjuvant chemotherapy. The patient was put in a treatment protocol consisting of cisplatin 80 mg/m^2^ on day 1 and 5-fluorouracil (5-FU) 800 mg/m^2^ from days 1 to 5. However, he was inadvertently administered with cisplatin 80 mg/m^2^ plus 5-FU 800 mg/m^2^ for consecutive 3 days, which fell upon Saturday, Sunday, and a national holiday in Japan. On day 4, which was Tuesday, the patient complained that he had hearing difficulty, and the cisplatin overdose was noted, and further chemotherapy was disrupted (Figure 1(a)). The patient was immediately transferred into a laminar flow clean room. Ototoxicity, nonoliguric renal failure, hepatic dysfunction, and acute pancreatitis were identified. Laboratory test revealed his BUN of 40.2 mg/dL, creatinine 1.99 mg/dL (175.9 *μ*M/L), AST 251 U/L, ALT 229 U/L, total bilirubin 0.6 mg/dL, amylase 178 U/L, and LDH 445 U/L. Hemodialysis and detoxification with sodium thiosulfate (STS) were performed on the same day and emergency plasma exchange was implemented on day 5 (Figure 1(a)). 

His plasma and urine total platinum concentrations were examined with flameless Zeeman atomic absorption spectrophotometry using Simultaneous Multielement Atomic Absorption Spectrometer 6000 (PerkinElmer. Inc., MA, USA). His plasma cisplatin concentration was 2,350 ng/mL after a cycle of hemodialysis and treatment with STS. On days 5 through 19, the patient underwent plasma exchange seven times and his plasma cisplatin concentration decreased to 110 ng/mL (Figure 1(a)). It was noted that his plasma cisplatin concentration was abruptly decreased after 2 cycles of plasma exchange; however, despite daily plasma exchange conducted, an increase of cisplatin concentration was observed twice, on days 8 and 10 (Figure 1(a)). 

His cisplatin excretion in urine was 4.8 mg/day on day 6. Of note, on day 15, when his plasma cisplatin concentration dropped below 180 ng/mL, cisplatin excretion in his urine yet persisted from 1.5 mg/day to 1.8 mg/day. On day 12, severe leukocytopenia occurred and the administration of granulocyte colony stimulating factor (G-CSF) was implemented. Leukopenia was noted on days 10–13 with WBC counts of ~2,000/mL and slowly worsened afterward. On day 14, he developed high fever with infectious focuses unknown and his granulocyte counts were of ~10/*μ*L, which persisted over 3 days despite the G-CSF administration (Figure 1(a)). Administration of broad-spectrum antibiotics (vancomycin and meropenem) was begun and his fever resolved by day 21. The patient was kept on fasting until day 19 because of mucositis that was thought to have resulted from cisplatin overdose and bacterial infection. 

After undergoing seven cycles of plasma exchange, his creatinine levels fell to 1.8 mg/dL (159.1 *μ*M/L) and his creatinine clearance got stabilized at 35 mL/minute. His serum levels of AST, ALT, and amylase were 240 U/L, 280 U/L, and 527 U/L, respectively, as examined on day 5; however, they became normal by day 10. He slowly recovered from his initial hearing loss, and after a month he subjectively did not perceive distinct ototoxicity. However, when his auditory acuity was evaluated, a significant acuity reduction was noted at high frequency ranges. His left/right auditory acuity levels were 20/35, 40/30, 30/30, 60/55, and 80/75 dB at 500, 1,000, 2,000, 4,000, and 8,000 Hz (normal auditory acuity levels are between 0–20 dB at each range: the greater the value, the more compromised the hearing acuity). 

His general conditions slowly but steadily improved without any further life-threatening complications arising from the cisplatin overdose and he was transferred into a general ward on day 28. Then, he was discharged later because of the eating disorders due to an esophageal stricture.

## 3. Discussion

Toxicities of cisplatin include emesis, nephrotoxicity, neurotoxicity, hearing loss, visual impairment, cholestasis, gastrointestinal disturbances, and bone marrow suppression [2]. The most serious complication is nephrotoxicity, which may result in irreversible renal failure [9, 10]. Patients inadvertently receiving less than 300 mg/m^2^ of cisplatin reportedly often recover, whereas overdoses exceeding 400 mg/m^2^ frequently result in death [2–7, 9, 11] (Table 1). As the toxicity of cisplatin is dose-dependent, early elimination of the drug from plasma should be critical in the management [12]. 

Reportedly, most of the platinum in the blood plasma is bound to proteins within a few hours after intravenous administration [4, 13]. The binding of cisplatin to proteins reduces urinary excretion of platinum and causes deposition of platinum in tissues. Binding of cisplatin to proteins and enzymes is generally believed to be the cause of its side effects, especially ototoxicity and nephrotoxicity. The protein-bound form cisplatin cannot be removed by hemodialysis [2, 4, 8, 14, 15]. Thus, hemodialysis is not effective in removing the protein-bound platinum; however, plasma exchange has been thought to be efficacious in treatment of cisplatin overdose. Indeed, in the present case, the plasma cisplatin concentration was as high as 2,350 ng/mL after one cycle of hemodialysis on day 4, while the plasma cisplatin concentration had decreased to 360 ng/mL after two cycles of plasma exchange (Figure 1(a)). Paradoxically, an increase of plasma cisplatin concentration was observed twice, on days 8 and 10 despite of daily plasma exchange conducted. These results suggest that cisplatin deposited in tissues and intracellular cisplatin [2, 6] were being continuously released to plasma. It is noteworthy that afterwards his plasma cisplatin concentration slowly but constantly decreased. It is argued as to how many cycles of plasma exchange are required to sufficiently decrease cisplatin to nontoxic levels. Therefore, we believe that early and continuous plasma exchange is useful in the management of cisplatin overdose. 

A number of thiols, including N-acetylcysteine, STS, and mesna, all of which bind to circurating reactive cisplatin derivatives, have been studied as chemoprotectants [7, 9]. These protectants are given before or during the administration of cisplatin.

In the present case, STS was administered on day 4; however, the efficacy of the administration in the present case is unclear [11]. Erdlenbruch et al. demonstrated that STS administrated 70 hours after an overdose had an effect in improving renal functions [7]. Nevertheless, there is no or little evidence that chemoprotectants can reverse hearing loss [16]. Moreover, it is of note that the use of chemoprotectant alone may impose overload to the kidney of patients since the elimination of cisplatin mostly occurs through the kidney, whose functions may have already been compromised by the toxicity of the agent.

As shown in Figure 1(b), the platinum clearance of the patient, which was calculated as platinum excreted per minutes divided by plasma platinum concentration, approximately correlated with creatinine clearance (Ccr). Significant amounts of platinum were excreted in the urine. While the plasma cisplatin concentration was as low as <180 ng/mL, the amounts of cisplatin excreted into urine were persistently >1.5 mg/day after Ccr was improved. Thus, in removing cisplatin as quickly as possible, sufficient hydration should be continued and Ccr levels should be cautiously monitored even after plasma cisplatin concentrations became apparently within or close to normal ranges. 

In the present case, we withheld the use of G-CSF until day 12, when the patient developed leucopenia. It is argued as to whether the administration of G-CSF should be implemented as soon as cisplatin overdose is revealed [6]. It is possible that stimulating hematopoietic cells to proliferate in the presence of toxic agents results in more substantial damage of such cells. It is known that certain anticancer agents such as cytarabine exert greater toxicity to granulocytes and granulocytic tumor cells when used with G-CSF [17]. Antiviral activity against human immunodeficiency virus of a nucleoside analogue, azidothymidine, is also potentiated in macrophages/monocytes when such cells are stimulated by granulocyte-macrophage-colony stimulating factor (GM-CSF) [18]. Another reason we withheld the use of G-CSF in the present case was that the patient had sufficient numbers of granulocytes and no signs of infections for a week after cisplatin overdosing, and we thought the administration of G-CSF was unnecessary. Indeed, G-CSF was started on day 12, when the patient had developed substantial leucopenia when his plasma platinum concentration had decreased from its peak to 210 ng/mL. 

Upon cisplatin overdose, the attempt of immediate, continuous, and sufficient removal of the drug is an important factor for the management of the overdose. In the present case, adverse events resulting from the overdose were successfully treated with vigorous plasma exchange combined with G-CSF administration and other supportive care. In order to prevent the recurrence of such an accident, it cannot be overemphasized that rigorous check systems and careful monitoring are essential when patients are treated with cytotoxic therapeutics.

## Figures and Tables

**Table 1:** Selected literature of cisplatin overdose; PE: plasma exchange; HD: hemodialysis. STS: sodium thiosulfate.

Authors	Dose of cisplatin	Treatment	Outcome
Schiller et al.	480 mg/m^2^	PE, HD	Alive, irreversible hearing loss
Chu et al.	280 mg/m^2^	PE, HD	Alive, irreversible hearing loss
Lagrange et al.	205 mg/m^2^	HD	Alive
Jung et al.	300 mg/m^2^	PE	Alive
Sheikh-Hamad et al.	400 mg/m^2^	N-acetylcysteine	Dead
Choi et al.	400 mg/m^2^	PE, HD	Alive
Erdlenbruch et al.	360 mg/m^2^	STS	Alive
Charlier et al.	750 mg/body	PE, HD, N-acetylcysteine	Dead
Hofmann et al.	225 mg/m^2^	PE	Alive
Our patient	240 mg/m^2^	PE, HD, STS	Alive
